# The relationship between the social media advertising content characteristics and the advertising effectiveness of female users: a moderated mediation model based on basic psychological needs

**DOI:** 10.3389/fpsyg.2026.1831184

**Published:** 2026-09-04

**Authors:** Yu Han

**Affiliations:** School of Foreign Studies, University of Science and Technology Beijing, Beijing, China

**Keywords:** advertising content characteristics, advertising effectiveness, moderated mediation effects, psychological need fulfillment, social media platforms

## Abstract

**Introduction:**

Based on self-determination theory, this study explores how the content characteristics of social media advertisements affect the advertising effectiveness of female users through meeting their basic psychological needs, and examines the moderating effect of platform environment.

**Methods:**

A total of 922 valid samples were obtained from female social media users aged 18-35 through an online questionnaire. The theoretical model was tested using confirmatory factor analysis and mediation, moderation, and moderation mediation effects analysis.

**Results:**

The authenticity, interactivity, and emotional resonance of advertisements are significantly indirectly correlated with brand loyalty, purchase intention, and information dissemination intention through the satisfaction of autonomy, competence, and belonging needs, respectively. Platform interactivity and community belonging can significantly enhance the relationship between advertising content characteristics and psychological needs satisfaction, but the moderating effect is relatively limited.

**Discussion:**

Research shows that meeting basic psychological needs is an important psychological mechanism that connects the characteristics of advertising content with advertising effectiveness, and the perceived platform environment constitutes its situational boundary. The study expands the application of self-determination theory in the field of social media advertising and provides reference for optimizing advertising content and platform environment.

## 1 Introduction

As social media platforms gradually become important fields for brand communication, user interaction, and consumption decision-making, the content creation methods of online advertising are undergoing significant changes (Riva, 2025). Traditional online advertising relies heavily on one-way information delivery and visual attraction. By contrast, advertising content in the social media era embeds platform recommendation algorithms, user interactive behaviors, community relational networks, and emotional expression frameworks (Yang et al., 2026; Stafford, 2025). Advertising is no longer merely a medium for presenting product information; it has also become an important medium for users to understand brands, express attitudes, participate in interactions, and construct self-identity (Karimova et al., 2025). On social media platforms including WeChat, Weibo, TikTok, and Xiaohongshu, advertising content appears in multiple forms (such as graphic recommendations, short videos, influencer product promotion, live-stream sales, and platform feed push). Its communication effect depends on content attractiveness; it also closely links with users’ perceived authenticity of advertisements, participation accessibility, and generated emotional resonance.

In this context, female users have become an important research group in social media advertising studies (Spais et al., 2024b). On one hand, female users maintain high social media engagement in categories (e.g., beauty, apparel, healthcare, maternal and infant products, lifestyle, emotional consumption, and interest communities). They also encounter platform-recommended advertisements, influencer marketing content, and community commercial information more frequently. On the other hand, female-targeted advertisements cover far more than commodity recommendations. They often interweave with topics (like body aesthetics, lifestyle shaping, emotional expression, identity construction, and group evaluation) (Kim, 2025; Spais et al., 2024a). Therefore, responses of female users toward advertising content are not simple information acceptance processes. They reflect comprehensive perceptions covering authenticity, right of choice, comprehension ability, emotional connection, and community bonds. Accordingly, this study explores the psychological need fulfillment mechanism of female users under social media advertising scenarios; this can help to have a deeper understanding of the correlation between online advertising content creation modes and advertising effectiveness.

Existing studies discuss the formation mechanism of social media advertising effectiveness from multiple perspectives. For example, advertising authenticity is believed to help strengthen user trust in advertising information and brand expressions; interactivity can enhance user engagement and enthusiasm for information processing; emotional resonance reinforces users’ attitudinal recognition of advertising content and willingness to share advertisements. Meanwhile, platform environments shape users’ understanding and responses toward advertising content. Platforms with powerful interactive functions provide users with channels for comments, likes, reposts, feedback, and group discussions. Platform environments with strong community belongingness easily integrate advertising content into shared interests, group identities, and emotional connections. Nevertheless, current research still has several limitations. First, most studies focus on the direct relationship between advertising content characteristics and advertising effectiveness; the interpretations of intermediate psychological mechanisms are still insufficient. Second, research on female users sometimes remains at the level of consumer group segmentation, lacking in-depth discussion of gendered media contexts within social media advertising. Third, platform interactivity and community belongingness are mostly described as background conditions. However, how they reshape the relationships between advertising content characteristics and users’ psychological needs requires further empirical verification.

Self-determination theory (SDT) supplies valuable theoretical foundations to deal with the above research issues. The theory identifies autonomy, competence, and belonging as three universal basic psychological needs of individuals within social contexts. When individuals feel that their choices and judgments are respected, the need for autonomy is more likely to be met; when individuals feel that they can understand information, master content, and make judgments, the need for competence is more likely to be satisfied. The belonging need gets satisfied when individuals build connections with other people, groups, or shared values. When this theory is applied to social media advertising scenarios, it can be found that advertising authenticity correlates with autonomy fulfillment. This is because authentic, transparent, and non-manipulative advertising content makes users feel their independent judgments are respected. Advertising interactivity relates to competence fulfillment. Interactive functions, feedback channels, and participation opportunities enhance users’ sense of comprehension and control over advertising information. Advertising emotional resonance may be related to belonging fulfillment, because advertising content that relates to life experience, value recognition, and identity expression is more likely to evoke users’ emotional connections and group identifications.

Based on the above theoretical logic, this study constructs a moderated mediation model at the individual level titled “advertising content characteristics—basic psychological need fulfillment—advertising effectiveness.” Specifically, the study selects advertising authenticity, interactivity, and emotional resonance as variables of advertising content characteristics. Autonomy, competence, and belonging are used as variables of basic psychological need fulfillment. Brand loyalty, purchase intention, and information dissemination willingness represent advertising effectiveness variables. Platform interactivity and community belongingness are further introduced as moderating variables at the individual perceived level. Based on SDT and model simplicity, this study selects three core paths with the strongest theoretical explanatory power for empirical examination. In addition, platform interactivity and community belongingness in this study refer to subjective perceived variables of respondents for its main advertising browsing platforms, rather than objective structural variables of platforms. Therefore, the constructed model is an individual-level moderated mediation model instead of a cross-level or multilevel model.

This study collects data by using the online questionnaire survey method. Respondents are female users aged 18 to 35 who use social media and encountered social media advertising within the past month. Respondents answer questions based on one social media platform they use most frequently and where they view the largest number of advertisements in the past month. The setting guarantees clear corresponding objects for measurements of advertising content characteristics, perceived platform environments, psychological need fulfillment, and advertising effectiveness. A total of 922 valid questionnaires were finally collected. This study systematically validates the theoretical model by using reliability and validity tests, confirmatory factor analysis (CFA), common method variance (CMV) test, correlation analysis, mediating effect test, moderating effect test, and moderated mediation effect test. Since all data derive from cross-sectional self-reported questionnaires, research results mainly reflect statistical correlations and model path relationships without strict causal inference.

The contributions of this study are mainly reflected in three aspects.

- This study advances studies on social media advertising effectiveness from direct relationships between advertising content characteristics and user behavioral responses. It provides mechanistic explanations based on basic psychological need fulfillment. Meanwhile, it reveals how authenticity, interactivity, and emotional resonance connect with distinct advertising effectiveness variables via autonomy, competence, and belonging, respectively.
- Starting from female users’ practical experience of social media advertising exposure, this study discusses female-targeted advertisements within integrated contexts of gendered media communication, emotional consumption, and community interaction. This can avoid simply treating female users as ordinary market segmentation groups.
- The study introduces platform interactivity and community belongingness as perceptual boundary conditions at the individual level. This further proves that social media platform environments are not neutral backgrounds; they may alter the strength of relationships between advertising content characteristics and users’ psychological need fulfillment.

Through the above research, this study hopes to provide certain theoretical references and practical inspirations for content creation, platform advertising governance, and user experience optimization of female-oriented online advertising in the social media era. It should be noted that this study does not directly investigate or experiment with the manipulation of the advertising content creation process. Each variable reflects the subjective perception of female users regarding their previous exposure to advertisements. Therefore, the implications of this study regarding content design belong to inferences formed based on statistical associations and should be understood as preliminary practical references rather than directly verified content production principles.

## 2 Literature review

Early research on advertising effectiveness does not merely focus on instant clicks or purchase behaviors within social media contexts. It establishes relatively stable research threads covering advertising attitude, purchase intention, brand attitude, brand trust, brand loyalty, and word-of-mouth communication. Traditional research on advertising effectiveness generally holds that advertising information influences consumer evaluations of brands and products through cognitive responses, behavioral intentions, and emotional responses. After the arrival of the online advertising stage, concepts (such as advertising value, advertising credibility, perceived informativeness, entertainment, irritation, perceived personalization, advertising skepticism, persuasion knowledge, and electronic word-of-mouth) gradually became key variables for explaining digital advertising effectiveness. Among these concepts, advertising credibility centers on users’ judgment of the authenticity and reliability of advertising information. Perceived informativeness reflects whether advertisements deliver valuable product information. Entertainment indicates whether advertising content brings pleasant experiences. Irritation refers to whether advertisements trigger disturbance, aversion or resistance. The persuasion knowledge theory further states that users identify commercial motives behind advertisements and adjust their attitudes and behavioral responses according to their judgments of persuasive intentions. Therefore, advertising effectiveness is not determined solely by advertising exposure or visual appeal. It is jointly shaped by advertising credibility, information value, persuasive intention recognition, and users’ psychological responses (Chakraborty and Biswal, 2026). With the development of short video platforms, influencer marketing, and immersive digital technologies, advertising communication has gradually changed from one-way information output to interactive, relational, and experiential communication. In the influencer marketing scenario, advertising content is often spread by influencers’ personal image, lifestyle narrative, and fan relationship trust. The acceptance of advertisements by users depends on the product information itself; it is also influenced by the content authenticity, relationship trust, and the interactive atmosphere of communities (Kovalenko et al., 2026; Li et al., 2026). Meanwhile, immersive technologies (such as AR, VR, MR, and 3D display) have further strengthened the advertising experience attributes. Thus, users have a stronger sense of presence, participation, and emotional investment when exposed to advertisements. This indicates that the social media advertising effectiveness is no longer the outcome of advertising content alone, but rather the result of the combined effect of advertising content, platform mechanisms, and user psychology.

In recent years, short-video platforms, live-streaming platforms, influencer marketing, and immersive technologies have further transformed advertising communication modes. Under influencer marketing and live-streaming scenarios, advertising content spreads relying on communicators’ credibility, attractiveness, professionalism, lifestyle narratives, and fan relationships. Recent research on medical live streaming shows that live streamers’ credibility and attractiveness affect users’ trust in content and participation responses. This proves that social media advertising effectiveness depends on advertising content itself and on communicator characteristics and interactive scenarios. Meanwhile, research on static posts in influencer public welfare communication reveals that static content is not necessarily inferior to video content; its emotional retention, narrative accumulation, and value evocation can also drive user engagement. Such studies indicate that social media advertising effectiveness needs to simultaneously examine content formats, communicator credibility, platform interactive mechanisms, and users’ psychological experiences.

In the aspect of advertising content characteristics, authenticity, interactivity, and emotional resonance are three important dimensions in the current social media advertising research. Authenticity is mainly reflected in the credibility and transparency of advertising information and the consistency with users’ real experiences. Studies have shown that authentic advertisements can reduce users’ perception of commercial manipulation and enhance their trust in brands. Interactivity emphasizes users’ participation, feedback, and control experience during advertising contact, including comments, likes, shares, customized choices, and communication with brands or other users (Depounti and Natale, 2025; Berry, 2025). Higher interactivity can enhance users’ sense of participation and improve the acceptance of advertising content. Emotional resonance emphasizes the connection between advertising content and users’ emotions, values, life experiences, and identity cognition. Advertisements with emotional resonance are more likely to stimulate users’ positive attitude towards brands and promote information dissemination behavior (Wang et al., 2025). In terms of advertising content characteristics, this study focuses on three dimensions: authenticity, interactivity, and emotional resonance. These three dimensions are not proposed in isolation, and they maintain theoretical connections with well-established constructs in existing advertising research. Authenticity bears close links with advertising credibility, brand trust, advertising skepticism, and persuasion knowledge. When users perceive advertising content as authentic, transparent, and consistent with real-life experiences, their defensive psychology against commercial manipulation and exaggerated promotion may weaken. Thus, users are more likely to build trust and independent judgment accordingly. Interactivity relates to perceived informativeness, user engagement, feedback control, and social interaction. In social media advertising, users join the advertising communication process via comments, likes, shares, reposts, private messages, secondary creation, and personalized selections. Such participation not only increases advertising exposure frequency but also improves users’ understanding and processing ability of advertising information. Emotional resonance is related to entertainment, emotional arousal, identity construction, value recognition and electronic word-of-mouth. Advertisements that resonate with users’ life experience, group identity, or value positions more easily stimulate emotional recognition and voluntary sharing intentions. As a result, authenticity, interactivity and emotional resonance can be regarded as concrete manifestations of social media advertising content characteristics across three levels: credibility, participation and emotional connection. However, current studies still have some limitations in explaining the relationship between advertising content characteristics and advertising effectiveness. Most studies mainly verify whether authenticity, interactivity, and emotional resonance can directly improve advertising effectiveness. However, there is less in-depth explanation of why these content features can affect users’ brand attitudes and behavioral intentions (Voronin and Rafikova, 2025). Many studies answer “whether the advertising content is effective,” but the discussion on “what psychological mechanism does the advertising content play a role in” is still insufficient. Existing studies explain advertising effectiveness from multiple perspectives (e.g., advertising credibility, informativeness, entertainment, irritation, perceived personalization, brand trust, advertising skepticism, persuasion knowledge, and electronic word-of-mouth). However, there are still certain deficiencies. First, many studies focus on direct relationships between advertising content characteristics and advertising effectiveness. Explanations of how users’ internal psychological needs connect advertising content with behavioral responses remain insufficient. Second, numerous social media advertising research discuss interaction, influencers, communities and platform recommendations. Nevertheless, interpretations of the psychological mechanisms driving users’ willingness to accept, participate in, purchase from or share advertising content are scattered. Third, most existing literature concentrates on emerging platform phenomena. However, the integration of SDT, basic psychological need measurement, and classic advertising effectiveness constructs is still insufficient. Therefore, it is necessary to establish a clearer connection between classic theories and recent social media advertising research. SDT offers an important theoretical basis for explaining this process. According to this theory, individual behavior is directly driven by external stimuli; it is also impacted by the degree of internal psychological need fulfillment. In the context of advertising communication, whether users feel that their choices are respected, whether they can understand and control advertising information, and whether they can get a sense of connection from advertising content will affect their subsequent reactions to advertisements and brands (Yang et al., 2024). Therefore, the introduction of basic psychological need fulfillment into the study of advertising effectiveness helps to further reveal the internal mechanism of advertising content characteristics influencing consumer behavior. SDT provides an important theoretical foundation to interpret the above mechanisms. It emphasizes that autonomy, competence and belonging are the basic psychological needs that individuals generally possess in social contexts. Autonomy means individuals feel their personal choices and judgments receive full respect; competence emphasizes that individuals feel they can understand the environment, master information and take effective actions; belonging represents individuals sense a connection with other people, groups or shared values. When these psychological needs are met, individuals are more likely to develop positive attitudes, intrinsic motivation, and sustained participation. Mature measurement tools (such as the basic psychological need scale and frustration scale) offer solid empirical bases for measuring autonomy, competence, and belonging. Introduction of SDT into social media advertising research helps explain why advertising content characteristics form correlations with purchase intention, brand loyalty, and information dissemination willingness.

Judging from the specific action path, authenticity, interactivity, and emotional resonance are theoretically related to different psychological experiences, respectively. Authentic advertising content can reduce users’ defensive psychology against false propaganda and manipulative communication; these make it easier for users to form brand attitudes based on their own judgments (Sun and Qu, 2025; Luo et al., 2025). Advertising with strong interactivity can provide users with opportunities for participation and feedback, so that they can gain a higher sense of information control and ability in the process of advertising contact (Liu and Xu, 2025). Emotional resonance makes it easier for users to associate advertising content with their own emotions and social relationships by stimulating common experience, value recognition, and relationship connection. Therefore, advertising content characteristics does not directly and mechanically affect advertising effectiveness; instead, they may further affect brand loyalty, purchase intention, and information dissemination willingness by activating users’ internal psychological experience. From specific functional paths, advertising authenticity mainly relates to autonomy fulfillment. Authentic, transparent and non-manipulative advertising content reduces users’ defensive psychology against false promotion and commercial manipulation. Users more easily interpret advertising content based on their independent judgments. When users perceive advertisements respect their choices and judgments, their autonomy needs gain higher satisfaction and further link to brand loyalty. Advertising interactivity mainly correlates with competence fulfillment. Highly interactive advertisements provide opportunities for participation, feedback, comments, sharing, and information control. Users gain stronger capacities to comprehend and master advertising information during exposure (Phan et al., 2026a). When users feel capable of understanding advertising information and forming judgments, their competence needs are better fulfilled and further associate with purchase intention. Advertising emotional resonance may mainly be related to belonging fulfillment. When advertising content can be connected with users’ emotions, life experience, value recognition or self-perception, users more easily feel understood, accepted, or bonded with a specific group. Belonging needs are satisfied accordingly and further linked to information dissemination willingness (Phan et al., 2026b). It should be clarified that this study does not claim that authenticity, interactivity, and emotional resonance exclusively correspond to one single psychological need. Three primary paths are selected for empirical testing based on theoretical focus and model parsimony. The platform environment is an important situational factor that affects the above psychological mechanism (Liang et al., 2025; Jiang et al., 2025). Social media platforms not only undertake the function of advertising distribution; they also affect the interpretation of advertising content through interaction rules, recommendation mechanisms, community atmosphere, and user relationship structure. In a platform with high interactivity, users can participate in the advertising communication process through comments, sharing, private messages, secondary creation, and real-time feedback; this may amplify the influence of advertising content on user psychological experience. In a platform with strong community belongingness, advertising content is more likely to be included in the discussion of common interests, identity, or common values, thus enhancing users’ acceptance and willingness to spread advertising content. Therefore, platform interactivity and community belongingness should not only be regarded as the background of advertising communication, but should be understood as important regulatory factors affecting the intensity of advertising content. The platform environment is an important situational factor that affects whether advertising content characteristics can be transformed into psychological need fulfillment. Social media platforms undertake advertising distribution functions. They also impact users’ interpretations of advertising content through interaction rules, recommendation algorithms, community atmospheres, and user relationship structures (Phan et al., 2026c). Under contexts with strong platform interactivity, functions (including comments, likes, reposts, feedback, private messages, and peer communication) enhance the participation and responsiveness of advertising content. Users more easily perceive and process advertising authenticity, interactivity, and emotional expressions. Under contexts with strong community belongingness, advertising content is readily incorporated into discussions centered on shared interests, group identities, and value recognition. Users obtain stronger senses of connection and belonging from advertising content. Therefore, this study defines platform interactivity and community belongingness as individual-perception moderating variables to explain variations in the strength of relationships between advertising content characteristics and basic psychological need fulfillment.

In addition, AI-driven intelligent promotion and algorithm recommendations are changing the operating logic of social media advertising. Intelligent recommendation systems can accurately target advertisements according to users’ browsing records, interactive behaviors, and interest preferences, thus improving the efficiency of advertisement matching (Shao et al., 2025; Shen and Wang, 2024). However, over-personalized and opaque algorithmic push may also cause users to worry about covert manipulation and privacy exposure, and weaken their trust in advertising content. Especially when the advertising content is highly matched with users’ interests, but the push logic lacks transparency, users may not interpret it as “useful information,” but may feel monitored, manipulated, or excessively tracked (Li et al., 2025; Ding et al., 2025). Therefore, the advertising effectiveness in the intelligent promotion environments relies on whether the recommendation is accurate; it also depends on whether the advertising content can maintain the user’s perception of independent choice and information control. This problem provides a new boundary condition for the study of social media advertising effectiveness; this also suggests that researchers need to pay attention to the balance between advertising efficiency and user psychological experience. Meanwhile, intelligent recommendations and platform algorithms change exposure modes of social media advertising. Perceived personalization improves matching degrees between advertising content and user interests; however, it also correlates with advertising skepticism, activation of persuasion knowledge, privacy concerns, and perceived manipulation. This study does not directly measure variables (such as advertising fatigue, push frequency, privacy worries, algorithm transparency, or perceived manipulation). Thus, this study only treats intelligent recommendation as a research background and future expansion direction, rather than a core variable within the empirical model. The core focus of this study remains how advertising content characteristics link to advertising effectiveness via basic psychological need fulfillment; it also focuses on how this process is moderated by users’ perceived platform interactivity and community belongingness. In addition, female users and identity-based media expression are also theoretical contexts that cannot be ignored in social media advertising research. Classic gender advertising studies state that advertisements deliver more than product information. Meanwhile, they construct gender meanings through body presentation, role arrangement, emotional narratives, and social expectations. Social media platforms further amplify such gendered expressions, as female-targeted advertisements frequently interweave with content covering beauty, apparel, healthcare, lifestyle, emotional consumption, and community evaluation. Pham and Phan (2025) indicated that identity-based communicators could influence user engagement and destination attractiveness through content expression and community bonds. Studies focusing on Vietnamese women’s online health medicine purchases also showed that female users’ digital consumption decisions were jointly affected by online experience, trust, and risk perception. Such studies prove that female social media users are not generic consumer segments; instead, they are advertising recipients within specific media expressions, platform relations, and social evaluation structures. Therefore, it is theoretically necessary for this study to examine the relationship between advertising content characteristics and psychological need fulfillment from the perspective of female users.

On the whole, existing studies have discussed the social media advertising effectiveness from the perspectives of advertising content, influencer marketing, platform interactivity, and intelligent recommendation. However, there are still some shortcomings. On the one hand, the study has paid more attention to the direct relationship between advertising content characteristics and advertising effectiveness. The explanation of psychological need fulfillment, a mediation mechanism, is still insufficient. On the other hand, platform environment variables are often treated as background conditions. The moderating effect of advertising content characteristics and psychological mechanisms has not been fully revealed. At the same time, the research on intelligent promotion mostly emphasizes accurate targeting and marketing efficiency. Insufficient attention has been paid to the decline of autonomy perception and users’ resistance. To sum up, the existing research has provided an important foundation for this study. Research on advertising effectiveness makes clear the importance of outcome variables (e.g., brand attitude, purchase intention, brand trust, brand loyalty, and electronic word-of-mouth). Online advertising research puts forward some key constructs, such as informativeness, entertainment, irritation, credibility, persuasion knowledge, advertising skepticism, and perceived personalization. The research of SDT and basic psychological need measurement offers a mature theory and measurement basis for explaining the internal psychological mechanism of users. Social media advertising, influencer marketing, and identity-based communication research show that platform interaction, content form, communicator credibility, and community relations affect users’ advertising response. However, existing studies still lack an analytical framework that can simultaneously integrate the perceived platform environment, advertising content characteristics, basic psychological need fulfillment, and female user contexts. Hence, this study constructs an individual-level moderated mediation model based on advertising content characteristics—basic psychological need fulfillment—advertising effectiveness. It focuses on testing how authenticity, interactivity, and emotional resonance separately form associations with brand loyalty, purchase intention, and information dissemination willingness via autonomy, competence, and belonging. Meanwhile, it further analyzes boundary effects exerted by platform interactivity and community belongingness on these paths.

## 3 Research model

This study focuses on whether advertising content characteristics are related to the brand attitudes and behavioral responses of female users and whether the basic psychological need fulfillment can statistically explain some of these correlations. This study revolves around one core theoretical question: in the context of social media, why are advertising content characteristics related to female users’ brand attitudes and behavioral responses, and through what psychological mechanisms? To answer this, the study constructs an individual-level moderated mediation model based on SDT; it incorporates advertising content characteristics, basic psychological need fulfillment, perceived platform environment, and advertising effectiveness into the same analytical framework. Among these, advertising authenticity, interactivity, and emotional resonance are used as advertising content characteristics variables; autonomy, competence, and belonging are basic psychological need fulfillment variables; brand loyalty, purchase intention, and information dissemination willingness serve as advertising effectiveness variables; while platform interactivity and community belongingness act as moderating variables at the level of individual perception.

SDT believes that autonomy, competence, and belonging are basic psychological needs that exist universally for individuals in different social situations. When these psychological needs are met, individuals are more likely to develop positive attitudes, intrinsic motivation, and sustained behavior engagement; when these psychological needs are weakened, individuals may experience resistance, alienation, or negative reactions. In the context of social media advertising, advertising is not merely product information or commercial stimulus; it is also a kind of media content with interactivity, symbolic meaning, and social relational attributes. Whether the advertising content is credible, allows user engagement and feedback, or can trigger emotional and value recognition may all be related to the extent to which users’ psychological needs are fulfilled; it is further associated with advertising effectiveness. Therefore, based on SDT, this study forms the following basic analytical logic: advertising content characteristics → basic psychological need fulfillment → advertising effectiveness.

**Figure 1:**
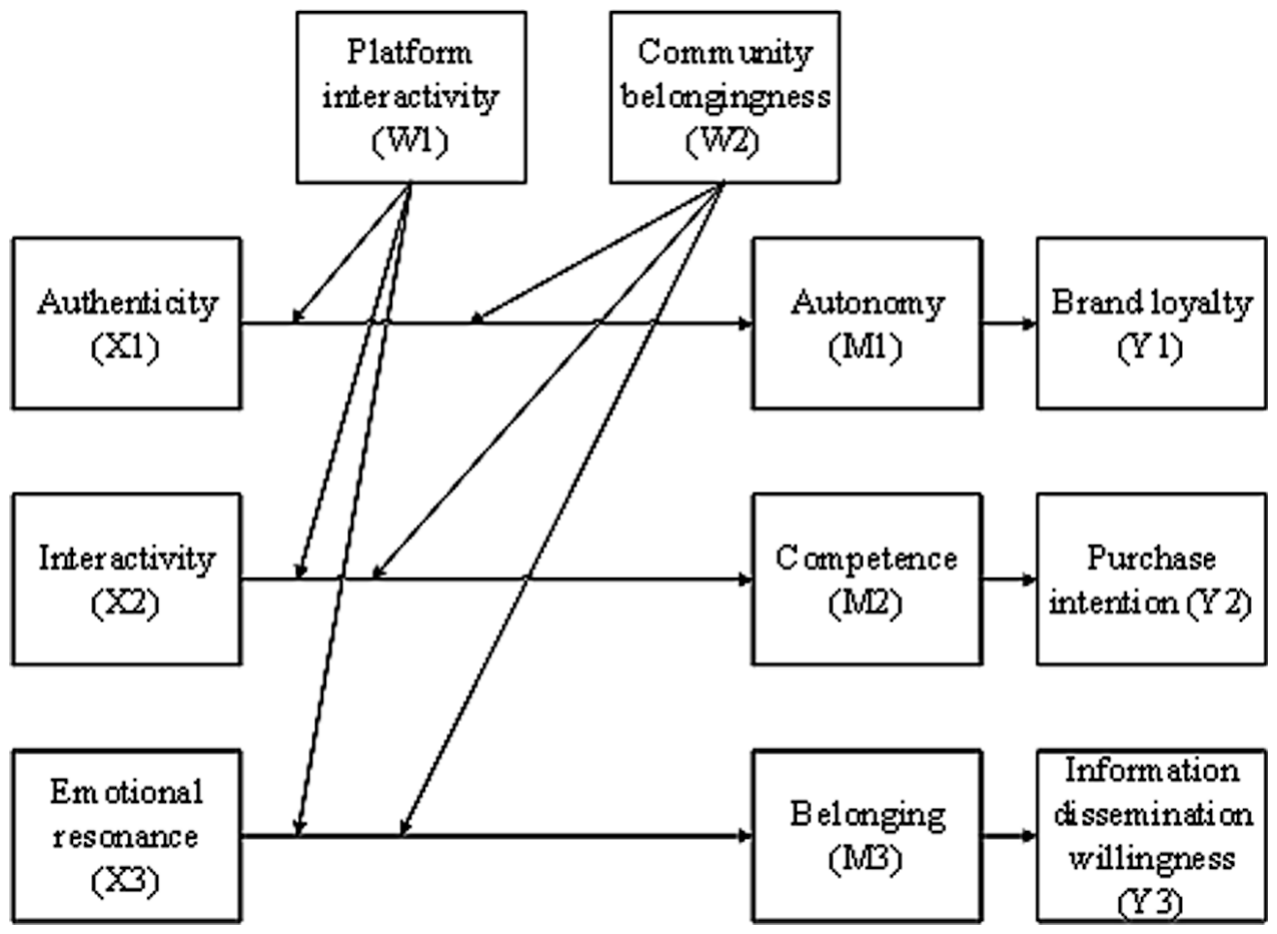
Theoretical model.

In this logic, this study takes the basic psychological need fulfillment as the mediating variable proposed theoretically to test whether it statistically connects advertising content characteristics perceived by users with advertising responses. This study further holds that platform interactivity and community belongingness are used as users’ subjective perception of social media platform environments; they may change the strength of the relationship between advertising content characteristics and basic psychological need fulfillment. The theoretical model of this study is shown in Figure 1.

This study selects authenticity, interactivity, and emotional resonance as the core advertising content characteristics. This is because the three can, respectively, reflect the activation methods of social media advertising content on users’ psychological experience from different aspects. Firstly, authenticity is mainly reflected in the consistency between advertising information and users’ real experiences, as well as whether advertising content is perceived as transparent, authentic, and non-manipulative. According to SDT, when users feel that their judgments and choices are respected, their autonomy needs are more likely to be met. Therefore, authentic, transparent, and non-manipulative advertising content are more likely to be associated with an enhanced perception of user autonomy. Secondly, interactivity emphasizes that advertising content provides users with opportunities to participate, feedback, comment, share, and express their opinions. Advertisements with strong interactivity can enhance users’ ability to understand, control, and process information, which is more likely to be related to competence fulfillment. Finally, emotional resonance represents the connection between advertising content and users’ emotions, life experiences, value recognition, and self-perception. When advertising content can make users feel understood, accepted, or shared with others, their belonging needs are more likely to be satisfied. It should be noted that this study does not deny that authenticity, interactivity, and emotional resonance may be cross-associated with multiple psychological needs simultaneously. For example, authenticity may also be related to brand trust and the experience of belonging; interactivity may also be linked to the sense of autonomous choice and relationship connection; emotional resonance may also be related to brand attitude and purchase intention. Based on theoretical focus and model simplicity, this study selects three main paths with the greatest explanatory power for testing. They are authenticity—autonomy—brand loyalty, interactivity-competence—purchase intention, and emotional resonance—belonging—information dissemination willingness.

Therefore, this study holds that advertising content characteristics do not directly and solely determine user attitudes and behaviors. Instead, it may form an indirect association with advertising effectiveness through different psychological need fulfillment paths.

In the context of social media advertising, autonomy, competence, and belonging correspond, respectively, to different types of advertising effectiveness. Autonomy fulfillment emphasizes that users feel their choices, judgments, and preferences are respected; this helps them form positive evaluations and continuous attention to the brand, thereby being related to brand loyalty. Competence fulfillment indicates that users can understand advertising information, master product content, and form judgment ability; this facilitates further understanding or trying the advertised products, thus being related to purchase intention. Belonging fulfillment means that users obtain emotional connection, identity construction, and group identification through advertising content; this helps to generate the willingness to like, comment, share, or recommend advertising content, thereby being associated with the information dissemination willingness. Thus, basic psychological need fulfillment constitutes the mediating mechanism between advertising content characteristics and advertising effectiveness. Meanwhile, social media platforms are not a completely neutral communication background. Users’ perception of platform interactivity and community belongingness may change the strength of the relationship between advertising content characteristics and psychological need fulfillment. Specifically, when users perceive that the platform has a strong interactive function, authenticity, interactivity, and emotional expression in advertising content are more likely to be activated through the mechanisms of comments, sharing, feedback, and participation. Hence, the associations between advertising content characteristics and psychological need fulfillment can be strengthened. When users perceive that the platform community has a strong sense of belonging, advertising content is more easily incorporated into discussions of common interests, common values, and group identifications, thereby further strengthening the psychological need fulfillment experience. It should be emphasized that both platform interactivity and community belongingness in this study are subjective perceptual variables at the individual level, rather than platform-level variables formed based on the objective data. Therefore, this study constructs a moderated mediation model at the individual level, rather than a cross-layer model or a multi-layer model.

The proposed model mainly includes the following three types of paths:

1. The relationship between advertising content characteristics and basic psychological need fulfillment, that is, the relationship between authenticity—autonomy—interactivity and competence—emotional resonance—belonging.
2. The mediating relationships of basic psychological need fulfillment between advertising content characteristics and advertising effectiveness. Specifically, autonomy mediates the link between authenticity and brand loyalty; competence mediates the link between interactivity and purchase intention; belonging mediates the link between emotional resonance and information dissemination willingness.
3. The moderating effect of perceived platform environment on the relationships between advertising content characteristics and basic psychological need fulfillment. That is, platform interactivity and community belongingness alter the strength of the three above-mentioned paths of advertising content characteristics—psychological need fulfillment.

By constructing this model, this study aims to provide a theoretical framework for understanding the psychological response mechanisms of female users toward advertising content within social media contexts. The core focus of this study does not lie in simply judging whether advertising content generates effective outcomes. Instead, it explains how distinct advertising content characteristics connect with advertising effectiveness through various psychological need fulfillment paths. At the same time, it further analyzes the boundary effects played by the perceived platform environment.

This study further puts forward the following research hypotheses.

- H1a: Advertising authenticity is positively related to users’ autonomy fulfillment.
- H1b: Advertising interactivity is positively associated with user competence fulfillment.
- H1c: Advertising emotional resonance is positively correlated with users’ belonging fulfillment.
- H2a: Users’ autonomy fulfillment is positively related to brand loyalty.
- H2b: Users’ competence fulfillment has a positive correlation with purchase intention.
- H2c: Users’ belonging fulfillment is positively associated with information dissemination willingness.
- H3a: Autonomy fulfillment plays a mediating effect between advertising authenticity and brand loyalty.
- H3b: Competence fulfillment exerts a mediating effect between advertising interactivity and purchase intention.
- H3c: Belonging fulfillment mediates the relationship between advertising emotional resonance and information dissemination willingness.
- H4a: Platform interactivity positively moderates the relationship between advertising authenticity and autonomy fulfillment.
- H4b: Platform interactivity positively moderates the relationship between advertising interactivity and competence fulfillment.
- H4c: Platform interactivity positively moderates the relationship between advertising emotional resonance and belonging fulfillment.
- H5a: Community belongingness positively moderates the relationship between advertising authenticity and autonomy fulfillment.
- H5b: Community belongingness positively moderates the relationship between advertising interactivity and competence fulfillment.
- H5c: Community belongingness positively moderates the relationship between advertising emotional resonance and belonging fulfillment.
- H6a: Platform interactivity moderates the indirect relationship between advertising authenticity and brand loyalty via autonomy fulfillment.
- H6b: Platform interactivity moderates the indirect relationship between advertising interactivity and purchase intention via competence fulfillment.
- H6c: Platform interactivity moderates the indirect relationship between advertising emotional resonance and information dissemination willingness via belonging fulfillment.
- H7a: Community belongingness moderates the indirect relationship between advertising authenticity and brand loyalty via autonomy fulfillment.
- H7b: Community belongingness moderates the indirect relationship between advertising interactivity and purchase intention via competence fulfillment.
- H7c: Community belongingness moderates the indirect relationship between advertising emotional resonance and information dissemination willingness via belonging fulfillment.

In addition, although advertising interactivity and perceived platform interactivity both relate to interaction, they differ in evaluation targets, conceptual scopes, and theoretical functions. Advertising interactivity takes specific advertising content that users have accessed as the evaluation object; it mainly reflects whether advertisements invite user responses, support information exploration, allow preference expression, and promote communication related to advertisements or brands. Thus, it belongs to the advertising content characteristic variable. Perceived platform interactivity is evaluated based on the overall environment of the social media platform mainly used by users; it emphasizes responsiveness, two-way nature, and continuity of platform communication and belongs to a general perceptual variable of users toward the advertising exposure context. Accordingly, advertising interactivity answers the question of “to what extent the advertising content itself supports user engagement”; perceived platform interactivity addresses “to what extent the platform environment where advertisements are placed sustains continuous, two-way and timely communication.” In the model, the former acts as a predictor linked to competence need satisfaction, and the latter is used as a contextual boundary variable for testing whether the strength of relationships between advertising content characteristics and basic psychological need fulfillment changes due to the perception of the platform environment. Therefore, although there may be a certain correlation between the two, they are not the same construct. The proposed research hypotheses are falsifiable and cannot be automatically confirmed merely upon completion of model estimation. For direct effects or moderating effects, if the estimated coefficient does not reach statistical significance, the direction is opposite to the hypothesis, or the confidence interval (CI) contains 0, the corresponding hypothesis is not supported. For the mediating effect, if the Bootstrap CI of the indirect effect contains 0, the mediating hypothesis is not supported. For moderated mediation effects, if the Bootstrap CI of the moderated mediation index contains 0, the corresponding hypothesis is not supported. Furthermore, even if the effect reaches statistical significance, if the standardized coefficient is extremely small, the incremental explained variance is limited, or the results in the robustness test are unstable, it also weakens the substantive support of the model.

## 4 Research design and methodology

### 4.1 Data sources and sample selection

An online questionnaire survey collects data for this study. Questionnaire data consist of subjective evaluations provided by respondents based on their personal social media usage and advertising exposure experiences. The purpose of this study does not involve estimating overall proportional parameters for the entire population of female social media users. It tests statistical correlations and psychological mechanisms among social media advertising content characteristics, basic psychological need fulfillment, perceived platform environment, and advertising effectiveness. Therefore, this study adopts a restricted voluntary online sampling method for sample collection. This method suits exploratory research and mechanism testing, yet interpretations of its conclusions are limited to the sample features and research context. Results cannot be directly generalized to all female social media users.

Questionnaires are distributed via online survey platforms. Sample recruitment channels include university alumni communities, female interest groups, and public or semi-public sharing links posted on social media platforms (such as WeChat, Weibo, TikTok, and Xiaohongshu). Restrictions on repeated submissions from the same device or account are set within the questionnaire system to reduce interference from duplicate and invalid responses. Duplicate, abnormal, and low-quality questionnaires are further identified during data cleaning. The opening page of the questionnaire displays research introductions and informed consent prompts. Respondents receive clear notification that all data from this study are solely used for academic analysis. The questionnaire adopts an anonymous format. No personally identifiable information, including names, phone numbers, ID numbers, and precise geographic locations, is collected. Respondents gain access to the formal questionnaire only after reading the research statement and confirming voluntary participation. The questionnaire takes an estimated 6 to 8 min to complete. The survey contains no experimental intervention, behavioral manipulation, medical intervention, sensitive physiological data collection, or risky stimuli. This study received ethical approval from the Medical Ethics Committee of Beijing Anzhen Hospital with approval number KS2025026. This ethical clearance applies to the anonymous, low-risk online questionnaire implemented in this study.

In order to ensure that the sample matches the research topic, screening questions are set at the beginning of the questionnaire. Respondents must meet the following conditions:

(1) The gender is female;
(2) The age is between 18 and 35 years old;
(3) Have experienced using social media within the past month;
(4) Have been exposed to advertising content on social media platforms within the past month;
(5) Be capable of conducting subsequent evaluations around a major social media platform that is frequently used and exposed to advertisements.

Questionnaires failing to meet the above criteria are excluded from formal analysis. The age range is restricted to 18–35 years old for two primary reasons. On the one hand, female users of this age group are an important group exposed to social media advertising, short-video content, influencer marketing, and platform recommendation ads. On the other hand, there are differences among diverse generational groups in terms of media usage habits, advertising exposure patterns, interactive behaviors, and expressions of psychological needs. Limiting the age range helps to reduce the interference from generational heterogeneity on model testing; this makes the samples more suitable for verifying the advertising psychological mechanism proposed in this study. This age restriction does not mean the conclusions apply to all age groups of female users, and cautious generalization is required.

To prevent ambiguous evaluation objects caused by respondents answering based on mixed cross-platform experiences, respondents select one social media platform with the highest monthly usage frequency and most frequent advertising exposure before starting formal questions. All subsequent items measuring advertising content characteristics, platform interactivity, community belongingness, basic psychological need fulfillment, and advertising effectiveness ask for evaluations based solely on advertising experiences from this primary selected platform. Therefore, the platform distribution in this study reflects respondents’ main evaluation platforms selected in the questionnaire, rather than their full scope of social media usage. Considering that users commonly use multiple platforms simultaneously, the platform distribution is not interpreted as exclusive user affiliation; meanwhile, causal comparisons across different platforms are not conducted. Platform interactivity and community belongingness are defined as individual-level perceived platform environment variables instead of platform-level variables derived from objective platform data. Thus, all subsequent model analyses are carried out at the individual level.

**Figure 2:**
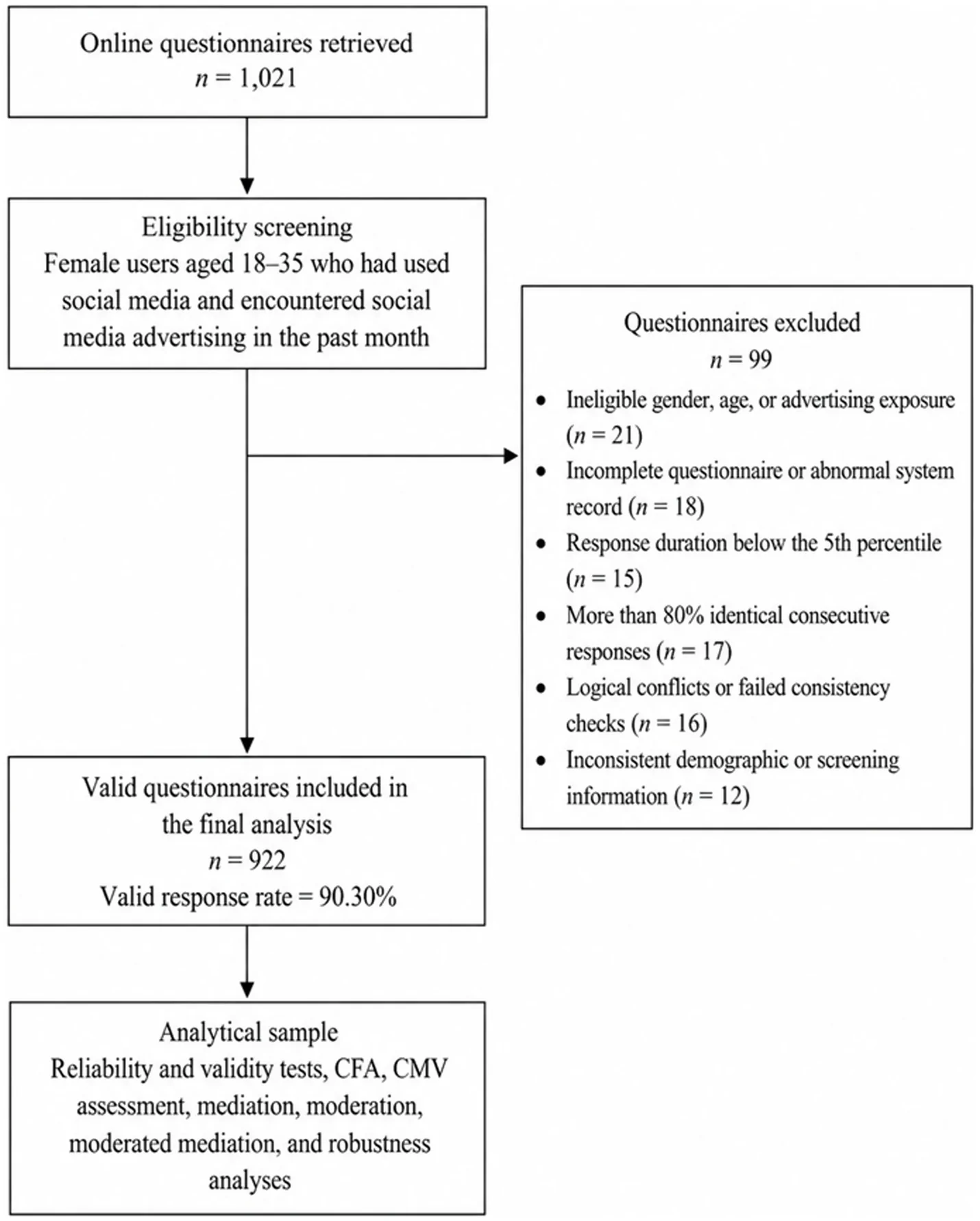
Sample selection flowchart.

A total of 1,021 questionnaires were retrieved in this study. In order to improve the data quality, this study conducts multiple rounds of screening on the original questionnaires. Six exclusion criteria are applied. First, questionnaires that do not meet the exposure conditions of gender, age, and social media advertising are removed. Second, the samples with missing or incomplete formal questionnaires in the core measurement items are excluded. Third, questionnaires with response durations below the 5th percentile of the full sample are eliminated, as the completion time is far shorter than normal reading and answering standards. Fourth, questionnaires where over 80% of responses to core scale items select identical single options are excluded. Fifth, questionnaires with obvious logical contradictions between reverse items and consistency check items are filtered out. Sixth, questionnaires with basic information inconsistent with screening criteria or abnormal response patterns are removed. The above standards are used to identify low-quality samples, while reasonable individual variations are retained. The specific process is presented in Figure 2.

After screening, a total of 99 invalid questionnaires were eliminated, and 922 valid questionnaires were obtained, with a valid recovery rate of 90.30%. The sample distribution is as follows:

(1) Social media platform distribution: WeChat, Weibo, TikTok, and Xiaohongshu users account for 45.6, 25.3, 15.8, and 13.3%, respectively.
(2) Geographic distribution: First-tier city users account for 39.7%; new first-tier city users account for 28.4%; second-tier and other city users account for 31.9%.
(3) Social media usage frequency: Heavy users spending over 3 h daily account for 33.5%; moderate users spending 1–3 h daily account for 48.6%; light users spending less than 1 h daily account for 17.9%.

The sample covers female users with diverse social media usage habits, city tiers, and income levels, providing sufficient data support for individual-level model testing. Basic sample demographic characteristics are shown below.

(1) Age distribution: Users aged 18–24, 25–30, and 31–35 account for 48.2, 32.7, and19.1%.
(2) Education level: Users with bachelor’s degrees or above account for 62.8%; users with college diplomas or below account for 37.2%.
(3) Income level: Users with monthly income below 5,000 RMB, between 5,000 and 10,000 RMB, and above 10,000 RMB account for 54.3, 34.5, and 11.2%.

Through the above-mentioned data sources, sample selection, and quality control processes, this study provides a relatively reliable data basis for the subsequent theoretical model verification. Meanwhile, this study acknowledges the potential self-selection bias inherent in voluntary online survey samples. Thus, limitations on sample generalizability are clarified in the conclusion section.

### 4.2 The correspondence between variable definitions and theories

This study centers on the psychological mechanism of advertising content characteristics—basic psychological need fulfillment—advertising effectiveness; it further examines the moderating effect of the perceived platform environment within this mechanism. According to the individual-level moderated mediation model constructed in Section 3, this study classifies variables into four categories: independent variables, mediating variables, moderating variables, and dependent variables. The independent variable consists of advertising content characteristics; the mediating variable refers to basic psychological need fulfillment; the moderating variable represents the perceived platform environment; the dependent variable stands for advertising effectiveness.

(1) The independent variable is advertising content characteristics, covering authenticity, interactivity, and emotional resonance.

Authenticity is the extent to which users perceive advertising content as authentic, credible, transparent, and consistent with real-life experiences. This variable mainly reflects whether advertising content reduces users’ perceptions of commercial manipulation, exaggerated promotion, and false expression.

Interactivity represents the extent to which users perceive advertising content as providing opportunities for participation, feedback, comment, sharing, selection, and communication. This variable mainly embodies whether advertising content enhances users’ sense of engagement and information control during advertising exposure.

Emotional resonance refers to the extent to which users perceive advertising content as connecting with their emotions, life experience, value recognition, or self-perception. This variable mainly reflects whether advertising content arouses users’ emotional connection and meaning identification.

(2) The mediating variables are basic psychological need fulfillment, including autonomy, competence, and belonging.

Autonomy means the degree to which users feel their personal choices, judgments, and preferences receive respect when exposed to advertising content. This study mainly uses it to explain the psychological mechanism between authenticity and brand loyalty.

Competence refers to the degree to which users feel they can understand advertising information, master relevant content, and develop the ability to make judgments. This study mainly employs it to interpret the psychological mechanism between interactivity and purchase intention.

Belonging represents the degree to which users gain emotional connection, a sense of being understood, and group identification through advertising content. It is mainly adopted in this study to interpret the psychological mechanism between emotional resonance and information dissemination willingness.

Based on SDT, autonomy, competence, and belonging are set as mediating psychological variables to explain the indirect relationships between advertising content characteristics and advertising effectiveness. It should be clarified that the three types of psychological needs are not exclusive to female users; they are universal psychological needs proposed in SDT. This study focuses on female users because the female consumer identity, emotional narratives, physical aesthetics, and community evaluation in social media advertising environments have obvious gendered communication features.

(3) Moderating variables are perceived platform environments, covering platform interactivity and community belongingness.

Platform interactivity refers to users’ perception of whether their primary platform supports functions including commenting, reposting, liking, feedback, private messaging, content participation, and peer communication. This variable reflects users’ subjective judgment of platform interactive functions and interactive atmosphere.

Community belongingness refers to users’ perception of whether platform communities have common interests, emotional support, group identities, and relationship connections. This variable demonstrates users’ subjective judgment of platform community atmosphere and the strength of group connections.

Special clarification is required: both platform interactivity and community belongingness are individual-level subjective perceptual variables, rather than group-level or platform-level variables formed based on objective platform data. Therefore, this study does not construct cross-layer models nor conduct causal comparisons at the platform level. The moderating effect of the perceived platform environment on the paths of advertising content characteristics—basic psychological need fulfillment is tested solely at the individual level.

(4) The dependent variable is advertising effectiveness, encompassing brand loyalty, purchase intention and information dissemination willingness.

Brand loyalty refers to the extent to which users develop sustained attention, trust, and repeated selection tendencies toward the brand featured in advertisements.

Purchase intention refers to the extent to which users consider learning more about, trying, or purchasing the advertised products or services.

Information dissemination willingness refers to the extent to which users are willing to like, comment on, share, repost, or recommend advertising content to other people.

The specific variable definitions and theoretical functions are exhibited in Table 1.

**Table 1:** Variable definitions and theoretical roles.

Variable category	Variable name	Symbol	Variable definition	Theoretical role
Independent variables	Advertising authenticity	X1	The extent to which users perceive the advertising content they have encountered as authentic, credible, transparent, and generally consistent with the actual products, services, or consumption experiences being presented.	A perceived advertising content characteristic and the predictor in the advertising authenticity–autonomy need fulfillment–brand loyalty path.
	Advertising interactivity	X2	The extent to which the advertising content encountered by users invites advertisement-related responses, supports further information exploration, enables users to express preferences or make choices, and facilitates two-way communication concerning the focal advertisement, brand, or product.	A perceived advertising content characteristic and the predictor in the advertising interactivity–competence need fulfillment–purchase intention path.
	Emotional resonance	X3	The extent to which users perceive that advertising content connects with their emotions, personal experiences, value recognition, or preferred lifestyles.	A perceived advertising content characteristic and the predictor in the emotional resonance–relatedness need fulfillment–advertising sharing intention path.
Mediating variables	Autonomy need fulfillment	M1	The extent to which users feel that their choices, judgments, and preferences are respected when they encounter advertising content and that they are not being forced or covertly manipulated.	A theoretically proposed mediating variable statistically connecting advertising authenticity with brand loyalty.
	Competence need fulfillment	M2	The extent to which users feel capable of understanding advertising information, evaluating the relevance of products or services, and making informed consumption judgments.	A theoretically proposed mediating variable statistically connecting advertising interactivity with purchase intention.
	Relatedness need fulfillment	M3	The extent to which users feel understood, accepted, or meaningfully connected with other people, interest groups, lifestyles, or shared values when encountering advertising content.	A theoretically proposed mediating variable statistically connecting emotional resonance with advertising sharing intention.
Moderating variables	Perceived platform interactivity	W1	Users’ overall perception of the responsiveness, reciprocity, and continuity of communication within their primary social media platform. It refers to the broader communication environment rather than the interactive design of a particular advertisement.	An individual-level perceived contextual variable that moderates the relationships between advertising content characteristics and basic psychological need fulfillment.
	Community belongingness	W2	Users’ perception that communities on their primary social media platform contain shared interests, common topics, emotional support, group identification, and relatively stable relational connections.	An individual-level perceived contextual variable that moderates the relationships between advertising content characteristics and basic psychological need fulfillment.
Dependent variables	Brand loyalty	Y1	The extent to which users are willing to maintain attention to, trust, prioritize, and continue following a brand after developing a positive response to its advertising content.	The advertising effectiveness outcome corresponding to autonomy need fulfillment.
	Purchase intention	Y2	The extent to which users are willing to learn more about, consider, try, or purchase the products or services presented in advertising content.	The advertising effectiveness outcome corresponding to competence need fulfillment.
	Advertising sharing intention	Y3	The extent to which users are willing to like, save, comment on, discuss, share, repost, or recommend advertising content to other people.	The advertising effectiveness outcome corresponding to relatedness need fulfillment.

To avoid confusion of theoretical concepts, this study further clarifies three main mechanism paths.

- Authenticity mainly corresponds to autonomy. Authentic, transparent, and non-manipulative advertising content makes users more likely to feel their judgments and choices are respected; this is further related to brand loyalty.
- Interactivity mainly corresponds to competence. Opportunities for participation, feedback, and information control improve users’ ability to comprehend advertising information and form judgments; this is further associated with purchase intention.
- Emotional resonance mainly corresponds to belonging. Emotional connection, value recognition, and life experience resonance strengthen users’ feelings of being understood, accepted, and group-bonded; this further links to information dissemination willingness.

Meanwhile, this study does not deny possible cross-correlations between authenticity, interactivity, emotional resonance, and other psychological needs or advertising effectiveness variables. For instance, authenticity may also correlate with trust, purchase intention, or sharing willingness; interactivity may also relate to perceived autonomy and relational bonds; emotional resonance may also associate with brand attitude and purchase intention. Guided by theoretical focus and model parsimony, three core paths with the strongest explanatory power are selected for empirical testing.

### 4.3 Questionnaire design, scale sources, and item settings

A structured questionnaire is adopted for data collection to guarantee standardized and operable measurement of research variables. The questionnaire is designed around four core variables: advertising content characteristics, basic psychological need fulfillment, perceived platform environment, and advertising effectiveness. All measurement items are contextually revised to fit social media advertising exposure scenarios, so that respondents can deliver evaluations based on their primary advertising browsing platform. The questionnaire consists of three sections: screening questions, demographic questions, and core measurement items.

The first section is screening questions. It verifies whether respondents meet the subject criteria, covering gender, age, monthly social media usage experience, and monthly exposure to social media advertising. The second section collects basic demographic information, including respondents’ age, education level, monthly income, city tier of residence, social media usage frequency, and primary advertising browsing platforms. The third section involves core measurement items that measure advertising authenticity, advertising interactivity, emotional resonance, autonomy, competence, belonging, platform interactivity, community belongingness, brand loyalty, purchase intention, and information dissemination willingness.

All core measurement items are rated on a five-point Likert scale, where 1 stands for strongly disagree and 5 stands for strongly agree. The score of each variable is the average value of its corresponding items. Higher scores indicate stronger perceived levels or behavioral tendencies of respondents toward the variable. A total of 36 core measurement items is set to cover 11 latent variables. To improve measurement transparency, this study fully lists all items, item numbers, corresponding variables, and primary reference sources in Appendix 1.

In terms of scale sources, well-established scales from advertising communication, consumer behavior, social media interaction, and SDT are prioritized and appropriately revised to adapt to social media advertising exposure contexts. Specifically, items for advertising authenticity mainly draw on studies concerning advertising credibility, perceived authenticity, and information transparency. Items for advertising interactivity reference scales related to perceived social media interaction, user engagement, and platform feedback mechanisms. Items for emotional resonance are derived from research on emotional arousal, value recognition, identity connection, and advertising resonance. Items measuring autonomy, competence, and belonging undergo contextual revision based on the basic psychological need fulfillment scale rooted in SDT. Items for platform interactivity and community belongingness refer to literature on social media platform environments, online community participation, and community identification. Items for brand loyalty, purchase intention, and information dissemination willingness adopt common attitudinal and behavioral intention measurement approaches widely used in advertising effectiveness and consumer behavior research.

In order to ensure rigorous scale adaptation, this study develops and revises the items in accordance with the standardized process. First, mature scales and frequently used measurement items relevant to the research variables are sorted out via literature screening; an initial item pool is built according to variable definitions. Second, original English items are translated into Chinese; item expressions are adjusted to fit social media advertising scenarios for respondents’ evaluations of their primary advertising browsing platform. Third, the research team conducts group discussions on all items, focusing on consistency with latent variable definitions, elimination of conceptual overlap, ambiguous wording, and leading language. Next, a bilingual back-translation method verifies key items. One researcher completes the Chinese translation, and another bilingual researcher translates the Chinese items back into English. Back-translated versions are compared against original items to maintain consistent core meanings.

After initial item drafting, experts specializing in advertising communication, consumer behavior, and psychology are invited for expert review. The review focuses on matching degrees between items and latent variables, sufficiency of content coverage, clarity of language expressions, and difficulty levels for respondent comprehension. The research team revises items with overly broad descriptions, vague conceptual boundaries, or ambiguous interpretations based on expert feedback. A small-sample pretest is then carried out. Eligible respondents fill out the draft questionnaire and submit feedback on questionnaire length, item comprehension, option settings, and overall answering experience. According to pretest results, questionnaire wording, item sequence, and answering instructions are further optimized to finalize the official questionnaire.

In order to mitigate biases from item order effects and social desirability, the opening section of the official questionnaire clearly informs respondents that no right or wrong answers exist. Responses only need to reflect genuine personal feelings. Core measurement items from different variables are moderately interleaved, thus preventing mechanical repetitive answering caused by concentrated similar items. Consistency check items and reverse-worded items are embedded in the questionnaire, so as to assist in identifying low-quality responses and logically contradictory samples. The full list of questionnaire items is presented in Appendix 1. To test for potential common method bias, a marker variable independent of the core theoretical variable is set in the questionnaire to measure the respondents’ neutral preference for the general interface presentation method. This marker variable has no direct relationship with advertising content characteristics, basic psychological needs, platform environment perception, and advertising effectiveness in theory.

### 4.4 Data cleaning, missing value handling, and outlier screening

Before the formal modeling, this study cleaned and screened the raw questionnaire data. First, questionnaire completeness is examined. All core measurement items are set as mandatory responses within the survey system. Therefore, no missing values exist for core variables, including advertising content characteristics, basic psychological need fulfillment, perceived platform environment, and advertising effectiveness. A listwise deletion method handles a small number of samples with irregular demographic entries, inconsistent screening criteria, or incomplete questionnaires; such samples are excluded from subsequent analysis.

This study further clarifies the data exclusion criteria:

(1) Samples that do not meet the gender, age, or social media advertising exposure conditions.
(2) Samples with unfinished core items or abnormal system recording records.
(3) Samples with response durations below the 5th percentile of the full sample.
(4) Samples where over 80% of responses to core scale items select identical single options consecutively.
(5) Samples presenting obvious logical conflicts between reverse-coded items and positive items.
(6) Samples with contradictory answers to consistency check items.
(7) Samples with logically inconsistent basic demographic information and screening responses.

Furthermore, this study conducts a preliminary examination of the data distribution. By examining the mean, standard deviation (SD), skewness, and kurtosis of each core variable, it is determined whether there are serious skewness or kurtosis issues with the variable. Samples are filtered by combining standardized residuals, outlier tests, and abnormal response patterns to reduce the influence of outliers on model estimation. Samples that deviate drastically from the overall distribution and display abnormal response features are eliminated. Individual variations within reasonable ranges are retained. Cleaned data support subsequent reliability, validity, correlation analysis, mediating effect test, moderating effect test, and moderated mediation effect test.

### 4.5 Data analysis methods and model settings

Based on the data structure and research questions, this study adopts the analysis methods of mediating effect, moderating effect, and moderated mediation effect at the individual level. All data are collected from independent individual respondents. Both platform interactivity and community belongingness are subjectively perceived variables of respondents for their main advertising browsing platform, rather than objective platform-level variables. Therefore, all the core variables of this study enter the analysis model at the individual level. This study does not adopt cross-layer models or multi-layer linear models to avoid the mismatch between the platform variable levels and the actual measurement levels.

To improve the robustness of the estimation results, this study adopts the Bootstrap resampling method in the mediating effect and moderated mediation effect tests. The sampling number is set at 5000 times, and the 95% CI is reported. When the Bootstrap CI does not contain 0, it indicates that the corresponding indirect effect or moderated mediation effect reaches a statistically significant level. All continuous variables undergo mean centralization before constructing the interaction term to reduce multicollinearity risks.

Based on the theoretical model proposed in Section 3, this study specifically examines the following three sets of paths:

- Relationships among authenticity, autonomy, and brand loyalty. The study examines whether authenticity has an indirect correlation with brand loyalty via autonomy. Meanwhile, it explores whether platform interactivity and community belongingness moderate the relationship between authenticity and autonomy.
- Relationships among interactivity, competence, and purchase intention. This study investigates whether interactivity forms an indirect correlation with purchase intention via competence; at the same time, it also analyzes whether platform interactivity and community belongingness moderate the relationship between interactivity and competence.
- Relationships among emotional resonance, belonging, and information dissemination willingness. The study explores whether emotional resonance has an indirect correlation with information dissemination willingness via belonging; it also examines whether platform interactivity and community belongingness moderate the relationship between emotional resonance and belonging.

To improve the reproducibility of the model report, this study further clarifies the model settings of various statistical tests. The mediating effect test uses PROCESS Model 4 to test the three paths of X1-M1-Y1, X2-M2-Y2, and X3-M3-Y3, respectively. The moderating effect test employs PROCESS Model 1 to examine the moderating effects of W1 and W2 on the three X → M paths, respectively. The moderated mediation effect test adopts PROCESS Model 7 to examine whether W1 and W2 moderate the indirect relationship between advertising content characteristics via basic psychological need fulfillment and advertising effectiveness. Control variables include age, education level, monthly income, city tier, social media usage frequency, and primary advertising browsing platform. Detailed model configurations are listed in Table 2. Hypothesis judgment does not rely solely on *p*-values. It comprehensively considers the Bootstrap CI, coefficient direction, standardized effect sizes, changes in explained variance, and robustness under different model specifications.

**Table 2:** Model settings for mediating, moderating, and moderated mediation analyses.

Test type	Statistical model	Independent variables	Mediating variables	Dependent variables	Moderating variables	Control variables
Mediating effect	PROCESS Model 4	X1	M1	Y1	—	Age, education level, monthly income, city tier, usage frequency, and primary platforms
		X2	M2	Y2	—	Age, education level, monthly income, city tier, usage frequency, and primary platforms
		X3	M3	Y3	—	Age, education level, monthly income, city tier, usage frequency, and primary platforms
Moderating effect	PROCESS Model 1	X1	—	M1	W1/W2	Age, education level, monthly income, city tier, usage frequency, and primary platforms
		X2	—	M2	W1/W2	Age, education level, monthly income, city tier, usage frequency, and primary platforms
		X3	—	M3	W1/W2	Age, education level, monthly income, city tier, usage frequency, and primary platforms
Moderated mediation effect	PROCESS Model 7	X1	M1	Y1	W1/W2	Age, education level, monthly income, city tier, usage frequency, and primary platforms
		X2	M2	Y2	W1/W2	Age, education level, monthly income, city tier, usage frequency, and primary platforms
		X3	M3	Y3	W1/W2	Age, education level, monthly income, city tier, usage frequency, and primary platforms

### 4.6 Criteria for hypothesis evaluation and falsifiability

In order to avoid equating successful statistical model estimation with the validity of theoretical hypotheses, this study sets prespecified falsification criteria for hypotheses. The fact that the corresponding model can be estimated, or a certain coefficient reaches a conventional significant level, does not mean that the hypothesis is automatically supported. This study comprehensively considers the coefficient direction, statistical significance, Bootstrap CI, effect magnitude, changes in explained variance, and robustness under different model specifications. Table 3 summarizes scenarios in which direct effects, mediating effects, moderating effects, and moderated mediation effects are rejected or receive only weak substantive support.

**Table 3:** Falsifiability criteria and observed outcomes.

**Hypothesis type**	**Rejection criterion**	**Observed result**	**Current judgment**
Direct association	Coefficient is nonsignificant, opposite in direction, or the CI includes zero	All six coefficients were positive, ranging from 0.264 to 0.345	Not rejected in the current sample
Mediation	Bootstrap CI of the indirect effect includes zero	Three indirect effects ranged from 0.077 to 0.112; all CIs excluded zero	Not rejected in the current sample
Moderation	Interaction coefficient is nonsignificant, opposite in direction, or its CI includes zero	Six interaction coefficients ranged from 0.087 to 0.145; all CIs excluded zero	Not rejected, but effects were small
Conditional indirect effect	Conditional indirect effects do not change across moderator levels	Low-to-high increases ranged from 0.042 to 0.073	Not rejected in the current sample
Moderated mediation	Bootstrap CI of the moderated mediation index includes zero	Indices ranged from 0.025 to 0.047; all CIs excluded zero	Not rejected in the current sample
Robustness	Core estimates change direction or become unstable across alternative specifications	Maximum absolute change in indirect effects was 0.006	Estimates remained stable
Substantive relevance	Effects are statistically significant but practically negligible	Moderation ΔR^2^ ranged from 0.012 to 0.022	Statistical support retained, but practical magnitude interpreted cautiously
Overall model	Multiple core paths are nonsignificant, opposite in direction, or unstable	No core path met the prespecified rejection criteria	Model not rejected within the current sample and design

## 5 Experimental analysis

### 5.1 Reliability, validity, and measurement model verification

Before officially conducting the path test, this study tests the reliability, convergent validity, discriminant validity, and overall fitting of the measurement model. Among them, Cronbach’s *α* and combined reliability are used to evaluate internal consistency; average variance extracted and standardized factor loading evaluate aggregated validity; CFA was employed to test the fitting and discriminant validity of the 11-factor measurement model. The results of reliability and aggregated validity are illustrated in Table 4.

**Table 4:** Reliability and convergent validity test results.

Variable category	Latent variable	Number of items	Range of standardized factor loadings	Cronbach’s α	CR	AVE
Advertising content characteristics	Advertising authenticity (X1)	3	0.742–0.831	0.846	0.851	0.657
	Advertising interactivity (X2)	3	0.718–0.807	0.829	0.834	0.626
	Emotional resonance (X3)	3	0.759–0.842	0.861	0.864	0.680
Basic psychological need fulfillment	Autonomy need fulfillment (M1)	3	0.726–0.814	0.837	0.842	0.641
	Competence need fulfillment (M2)	3	0.705–0.796	0.812	0.817	0.598
	Relatedness need fulfillment (M3)	3	0.768–0.846	0.872	0.875	0.701
Perceived platform environment	Perceived platform interactivity (W1)	3	0.736–0.823	0.841	0.846	0.647
	Community belongingness (W2)	3	0.755–0.835	0.858	0.862	0.675
Advertising effectiveness	Brand loyalty (Y1)	4	0.721–0.831	0.881	0.884	0.656
	Purchase intention (Y2)	4	0.734–0.849	0.894	0.898	0.688
	Advertising sharing intention (Y3)	4	0.716–0.826	0.876	0.879	0.646

Table 4 shows that the Cronbach’s α value of every latent variable exceeds 0.800, indicating that the scale has good internal consistency. All latent variables have CR and AVE values above 0.800 and 0.500, with generally high standardized factor loadings. This demonstrates favorable composite reliability and convergent validity for each latent variable.

To further test the measurement model’s overall adaptability, this study uses CFA to test the 11-factor measurement model. The results are revealed in Table 5.

**Table 5:** Confirmatory factor analysis fit indices.

Model	χ^2^	df	χ^2^/df	CFI	TLI	RMSEA	SRMR
The 11-factor measurement model	1324.612	548	2.418	0.936	0.927	0.039	0.045
Recommended standard	—	—	< 3.000	> 0.900	> 0.900	< 0.080	< 0.080

Table 5 shows that the χ^2^/df of the 11-factor measurement model is 2.418, lower than 3. Comparative Fit Index (CFI) reaches 0.936, and Tucker-Lewis Index (TLI) is 0.927, both higher than 0.900. The Root Mean Square Error of Approximation (RMSEA) is 0.039, and the Standardized Root Mean Square Residual (SRMR) is 0.045, both lower than 0.080. This illustrates that the proposed measurement model exhibits good overall fit, and each latent variable can be distinguished relatively clearly.

In order to test the discriminant validity among latent variables, this study conducts the analysis using the Fornell-Larcker criterion. Table 5 lists the square root of AVE for each latent variable and Pearson correlation coefficients among the latent variables. Among them, diagonal values are the square root of AVE, and off-diagonal values stand for Pearson correlation coefficients of variables.

Table 6 reveals that the square root of AVE for each latent variable exceeds its correlation coefficients with all other latent variables. This proves that there is good discriminative validity among latent variables. After further verification using the Heterotrait-Monotrait Ratio (HTMT) method, the HTMT values among the variables range from 0.382 to 0.714, all below the critical threshold of 0.850. This further confirms that there is no serious conceptual overlap among latent variables.

**Table 6:** Discriminant validity test results.

**Variable**	**X1**	**X2**	**X3**	**M1**	**M2**	**M3**	**W1**	**W2**	**Y1**	**Y2**	**Y3**
X1	0.811										
X2	0.384	0.791									
X3	0.402	0.376	0.825								
M1	0.456	0.318	0.335	0.801							
M2	0.301	0.421	0.327	0.358	0.773						
M3	0.342	0.336	0.478	0.384	0.391	0.837					
W1	0.365	0.414	0.389	0.377	0.402	0.398	0.804				
W2	0.348	0.369	0.431	0.361	0.385	0.447	0.426	0.822			
Y1	0.392	0.311	0.343	0.438	0.356	0.379	0.342	0.365	0.810		
Y2	0.326	0.417	0.354	0.337	0.452	0.382	0.374	0.386	0.431	0.830	
Y3	0.345	0.336	0.462	0.351	0.376	0.486	0.389	0.421	0.397	0.424	0.804

In addition, to test whether there is a problem of multicollinearity among variables, this study calculates tolerance and Variance Inflation Factor (VIF) values for core independent variables, mediating variables, moderating variables, and interaction terms. The results are displayed in Table 7.

**Table 7:** Multicollinearity test results.

Variable	Tolerance	VIF
Advertising authenticity (X1)	0.661	1.513
Advertising interactivity (X2)	0.703	1.423
Emotional resonance (X3)	0.642	1.558
Autonomy need fulfillment (M1)	0.604	1.655
Competence need fulfillment (M2)	0.637	1.570
Relatedness need fulfillment (M3)	0.589	1.698
Perceived platform interactivity (W1)	0.534	1.873
Community belongingness (W2)	0.518	1.931
X1 × W1	0.487	2.053
X2 × W1	0.502	1.992
X3 × W1	0.476	2.101
X1 × W2	0.491	2.037
X2 × W2	0.508	1.969
X3 × W2	0.463	2.159

Table 7 indicates that the VIF values of every variable and interaction term are all lower than 3, and all tolerance values exceed 0.10. This indicates that there is no serious multicollinearity problem in the proposed model. Based on the above results, the questionnaire measurement instrument meets acceptable standards in terms of reliability, convergent validity, discriminant validity, and model fit. Thus, it supports subsequent path testing.

### 5.2 CMV control and test

Because all core variables of this study come from the same time point, questionnaire, and self-reported data collected from identical respondents, there may be CMV problems. In order to reduce the influence of CMV on the conclusions, this study takes control measures at the questionnaire design and statistical test stages, respectively. At the questionnaire design stage, this study uses anonymous responses, clear statements that no right or wrong answers exist, an interleaved item arrangement, and consistency check items to reduce social desirability bias and mechanical answering patterns. At the statistical test stage, three methods are used to diagnose CMV: Harman’s single-factor test, comparison between the single-factor CFA model and the multi-factor model, and the common method factor test.

Firstly, this study constructs a single-factor model and compares it with the theoretical 11-factor model. The results are plotted in Table 8.

**Table 8:** Test results of CMV.

Model	χ^2^	df	χ^2^/df	CFI	TLI	RMSEA	SRMR
Single-factor model	3882.314	565	6.872	0.621	0.598	0.091	0.104
Theoretical 11-factor model	1324.612	548	2.418	0.936	0.927	0.039	0.045
Difference	2557.702	17	—	0.315	0.329	0.052	0.059

Table 8 shows a poor fit for the single-factor model. Its χ^2^/df is 6.872; CFI and TLI fall far below 0.900; RMSEA and SRMR exceed recommended cutoffs. By contrast, the theoretical 11-factor model achieves satisfactory fit. This means that the data is not dominated by a single common method factor.

Subsequently, this study adds the common method factor to the theoretical model to further examine whether CMV substantially changes the model fit structure. The results are shown in Table 9.

**Table 9:** Test results of CMV.

Model	CFI	TLI	RMSEA	SRMR	ΔCFI relative to the theoretical model	ΔRMSEA relative to the theoretical model	Interpretation
Theoretical 11-factor model	0.936	0.927	0.039	0.045	—	—	Baseline measurement model
Model with a common latent method factor	0.947	0.936	0.035	0.039	0.011	0.004	Model fit improved modestly after the common method factor was added

Table 9 demonstrates that after adding the common method factor, the model fit indicators only show limited improvement. The CFI and RMSEA change by 0.011 and 0.004, neither reaching the degree of significantly altering the model’s explanatory structure. Meanwhile, the direction and statistical significance of core paths remain essentially unchanged after introducing the common method factor. Therefore, CMV poses no serious threat to the primary research conclusions.

### 5.3 Descriptive statistics and correlation analysis

Descriptive statistics and correlation analysis are conducted for all core variables. Table 10 reports the mean, SD, skewness, kurtosis, and Pearson correlation coefficients of each variable.

**Table 10:** Descriptive statistics and correlation analysis results.

**Variable**	**M**	**SD**	**Skewness**	**Kurtosis**	**X1**	**X2**	**X3**	**M1**	**M2**	**M3**	**W1**	**W2**	**Y1**	**Y2**	**Y3**
X1	4.001	0.684	−0.312	0.418	1										
X2	3.512	0.711	−0.186	0.227	0.384**	1									
X3	3.768	0.861	−0.265	0.306	0.402**	0.376**	1								
M1	3.487	0.692	−0.142	0.198	0.456**	0.318**	0.335**	1							
M2	3.694	0.865	−0.221	0.251	0.301**	0.421**	0.327**	0.358**	1						
M3	4.002	0.713	−0.336	0.482	0.342**	0.336**	0.478**	0.384**	0.391**	1					
W1	3.724	0.738	−0.205	0.236	0.365**	0.414**	0.389**	0.377**	0.402**	0.398**	1				
W2	3.806	0.752	−0.243	0.319	0.348**	0.369**	0.431**	0.361**	0.385**	0.447**	0.426**	1			
Y1	3.756	0.776	−0.214	0.275	0.392**	0.311**	0.343**	0.438**	0.356**	0.379**	0.342**	0.365**	1		
Y2	3.703	0.794	−0.198	0.241	0.326**	0.417**	0.354**	0.337**	0.452**	0.382**	0.374**	0.386**	0.431**	1	
Y3	3.681	0.803	−0.176	0.219	0.345**	0.336**	0.462**	0.351**	0.376**	0.486**	0.389**	0.421**	0.397**	0.424**	1

Table 10 shows that the mean values of each variable range from 3.487 to 4.002. This reflects moderately positive overall evaluations of advertising content characteristics, psychological need fulfillment, perceived platform environment and advertising effectiveness among respondents. The absolute values of skewness and kurtosis of each variable are all within an acceptable range; this indicates that the main variables do not have serious skewness or kurtosis issues, basically meeting the requirements for subsequent parameter analysis.

The results of correlation analysis show that authenticity is significantly positively correlated with autonomy; interactivity is significantly positively related to competence; emotional resonance is significantly positively associated with belonging. This indicates that the three core paths of advertising content characteristics—psychological need fulfillment proposed in the theoretical model have preliminary statistical support. In addition, autonomy and brand loyalty, competence and purchase intention, belonging and information dissemination willingness all exhibit remarkable positive correlations, laying preliminary foundations for subsequent mediation effect tests.

It should be clarified that correlation analysis only reflects statistical associations rather than strict causal relationships between variables. Therefore, this study primarily adopts terms (such as correlation, association, and indirect association) in the following result interpretations to avoid overstated causal inferences.

### 5.4 Mediation effect testing

PROCESS Model 4 is adopted for mediation effect tests to verify the mediating roles of basic psychological need fulfillment between advertising content characteristics and advertising effectiveness. The Bootstrap resampling method with 5,000 iterations computes the 95% CI. A statistically significant mediating effect is confirmed if the Bootstrap 95% CI excludes 0. Three mediating paths proposed in Section 3 are tested separately: authenticity—autonomy—brand loyalty, interactivity—competence—purchase intention, and emotional resonance—belonging—information dissemination willingness. The test results are depicted in Table 11.

**Table 11:** Mediation effect test results.

Mediation path	X → M β	M → Y β	Direct effect	Indirect effect	Total effect	Boot SE	Bootstrap 95% CI	Proportion mediated	Effect interpretation
Advertising authenticity → autonomy need fulfillment → brand loyalty	0.312***	0.276**	0.214**	0.086	0.300	0.026	[0.038, 0.141]	28.7%	Partial mediation
Advertising interactivity → competence need fulfillment → purchase intention	0.264**	0.290***	0.203**	0.077	0.280	0.025	[0.031, 0.128]	27.5%	Partial mediation
Emotional resonance → relatedness need fulfillment → advertising sharing intention	0.345***	0.325***	0.246***	0.112	0.358	0.030	[0.056, 0.174]	31.3%	Partial mediation

Table 11 presents significant positive relationships between authenticity and autonomy as well as autonomy and brand loyalty. Authenticity generates a significant indirect association with brand loyalty via autonomy. The indirect effect is 0.086, with the Bootstrap 95% CI of [0.038, 0.141] that excludes zero. Hence, H1a, H2a, and H3a receive empirical support.

There is a significant positive relationship between interactivity and competence as well as competence and purchase intention. Interactivity forms a significant indirect association between competence and purchase intention, with an indirect effect of 0.077. The Bootstrap 95% CI is [0.031, 0.128], excluding 0. Therefore, H1b, H2b, and H3b are supported.

There is a significant positive relationship between emotional resonance and belonging as well as belonging and information dissemination willingness. Emotional resonance has a significant indirect association between belonging and information dissemination willingness, with an indirect effect of 0.112. The Bootstrap 95% CI is [0.056, 0.174], excluding 0. Thus, H1c, H2c, and H3c are supported.

Overall, all three mediating paths achieve statistical significance. This proves that basic psychological need fulfillment exerts a prominent mediating effect between advertising content characteristics and advertising effectiveness. Among the three paths, the indirect effect of the emotional resonance—belonging—information dissemination willingness path is relatively larger. It indicates that within social media advertising contexts, emotional connection and value recognition more easily link to users’ voluntary sharing willingness through the sense of belonging.

### 5.5 Moderating effect testing

PROCESS Model 1 is utilized for moderation effect tests to examine whether the perceived platform environment moderates the relationships between advertising content characteristics and basic psychological need fulfillment. Platform interactivity and community belongingness are separately entered into the model as moderating variables. The core test focuses on the statistical significance of interaction terms. All continuous variables undergo mean centering before constructing interaction terms to reduce multicollinearity risks. The outcomes of the moderating effect test are summarized in Table 12.

**Table 12:** Moderating effect test results.

Moderating path	Moderator	Interaction β	SE	t	p	ΔR^2^	Bootstrap 95% CI	Incremental effect size	Statistical judgment
Advertising authenticity → autonomy need fulfillment	Perceived platform interactivity (W1)	0.118	0.025	4.720	<0.001	0.018	[0.052, 0.179]	Small, 1.8% additional variance	Supported in the current sample
Advertising interactivity → competence need fulfillment	Perceived platform interactivity (W1)	0.092	0.023	4.000	<0.001	0.014	[0.027, 0.146]	Small, 1.4% additional variance	Supported in the current sample
Emotional resonance → relatedness need fulfillment	Perceived platform interactivity (W1)	0.145	0.027	5.370	<0.001	0.022	[0.081, 0.213]	Small, 2.2% additional variance	Supported in the current sample
Advertising authenticity → autonomy need fulfillment	Community belongingness (W2)	0.102	0.025	4.080	<0.001	0.016	[0.041, 0.161]	Small, 1.6% additional variance	Supported in the current sample
Advertising interactivity → competence need fulfillment	Community belongingness (W2)	0.087	0.023	3.783	<0.001	0.012	[0.019, 0.138]	Small, 1.2% additional variance	Supported in the current sample
Emotional resonance → relatedness need fulfillment	Community belongingness (W2)	0.131	0.026	5.038	<0.001	0.019	[0.067, 0.196]	Small, 1.9% additional variance	Supported in the current sample

Table 12 reveals that platform interactivity exerts a significant positive moderating effect on all three X → M paths. The interaction term of authenticity and platform interactivity is significantly positive. This means that the positive association between authenticity and autonomy becomes stronger when users perceive high platform interactivity, supporting H4a. The interaction term of interactivity and platform interactivity is significantly positive. This indicates that when the platform’s interactive functions and interactive atmosphere are abundant, the positive relationship between interactivity and competence is more obvious, and H4b is supported. The interaction term of emotional resonance and platform interactivity is also significantly positive. This suggests that platform interactivity strengthens the relationship between emotional resonance and belonging, verifying H4c.

In the meantime, community belongingness produces significant positive moderating effects on all three X → M paths. The interaction term of authenticity and community belongingness is significantly positive. This indicates that the connection between authenticity and autonomy grows stronger under high community belongingness, supporting H5a. The interaction term of interactivity and community belongingness is significantly positive. This means that community belongingness reinforces the positive correlation between interactivity and competence, which supports H5b. The interaction term of emotional resonance and community belongingness is significantly positive. This illustrates that when users perceive strong emotional support and group identity within platform communities, emotional resonance more readily forms positive associations with belonging, verifying H5c.

It should be clarified that the moderation results do not imply unconditional positive effects brought by platform interactivity and community belongingness under all circumstances. The findings only indicate that higher platform interactivity and community belongingness correlate with stronger X → M path relationships within the current sample and model settings. The specific functions require cautious interpretation combined with platform environments, advertising content types and users’ perceptual patterns.

### 5.6 Moderated mediation effect testing

On the basis of validated mediating and moderating effects, PROCESS Model 7 is further applied to test moderated mediation effects. The test judges whether platform interactivity and community belongingness additionally moderate the indirect relationships from advertising content characteristics to advertising effectiveness through basic psychological need fulfillment. Indirect effects are calculated under three levels of each moderating variable: low, medium, and high. The low level is defined as one SD below the mean, the medium level is the mean, and the high level is one SD above the mean. A significant moderated mediation effect exists if the Bootstrap 95% CI of the moderated mediation index excludes 0. The test results are presented in Table 13.

**Table 13:** Moderated mediation effect test results.

Indirect path	Moderator	Low level	Indirect effect at low level	Mean level	Indirect effect at mean level	High level	Indirect effect at high level	Increase from low to high	Moderated mediation index	Bootstrap 95% CI
Advertising authenticity → autonomy need fulfillment → brand loyalty	Perceived platform interactivity (W1)	2.986	0.061	3.724	0.086	4.462	0.114	0.053	0.033	[0.012, 0.061]
Advertising interactivity → competence need fulfillment → purchase intention	Perceived platform interactivity (W1)	2.986	0.052	3.724	0.077	4.462	0.101	0.049	0.027	[0.009, 0.052]
Emotional resonance → relatedness need fulfillment → advertising sharing intention	Perceived platform interactivity (W1)	2.986	0.078	3.724	0.112	4.462	0.151	0.073	0.047	[0.019, 0.083]
Advertising authenticity → autonomy need fulfillment → brand loyalty	Community belongingness (W2)	3.054	0.064	3.806	0.086	4.558	0.109	0.045	0.028	[0.010, 0.055]
Advertising interactivity → competence need fulfillment → purchase intention	Community belongingness (W2)	3.054	0.055	3.806	0.077	4.558	0.097	0.042	0.025	[0.007, 0.049]
Emotional resonance → relatedness need fulfillment → advertising sharing intention	Community belongingness (W2)	3.054	0.082	3.806	0.112	4.558	0.146	0.064	0.043	[0.016, 0.078]

Table 13 shows that when platform interactivity acts as the moderating variable, the indirect effects of all three paths increase along with rising platform interactivity levels. For the authenticity—autonomy—brand loyalty path, the indirect effects under low, medium, and high platform interactivity are 0.061, 0.086, and 0.114, respectively. The moderated mediation index equals 0.033, and the Bootstrap 95% CI is [0.012, 0.061], excluding zero. Platform interactivity significantly moderates this indirect path, so H6a is supported. For the interactivity—competence—purchase intention path, the moderated mediation index is 0.027 and the Bootstrap 95% CI is [0.009, 0.052], verifying H6b. For the emotional resonance—belonging—information dissemination willingness path, the moderated mediation indicator is 0.047, and the Bootstrap 95% CI is [0.019, 0.083], which supports H6c.

When community belongingness is used as a moderating variable, the three indirect paths also show a similar trend. In the authenticity-autonomy-brand loyalty path, the moderated mediation index is 0.028, the Bootstrap 95% CI is [0.010, 0.055], and H7a is supported. For the interactivity-competence-purchase intention path, the moderated mediation index is 0.025, the Bootstrap 95% CI is [0.007, 0.049], verifying H7b. For the emotional resonance—belonging—information dissemination willingness path, the moderated mediation index is 0.043, and the Bootstrap 95% CI is [0.016, 0.078], supporting H7c.

On the whole, both platform interactivity and community belongingness significantly moderate the indirect relationships between advertising content characteristics and advertising effectiveness via basic psychological need fulfillment. The moderated mediation effect of the emotional resonance—belonging—information dissemination willingness path is relatively stronger. This demonstrates that emotional connection and community atmosphere exert prominent correlations with users’ voluntary sharing willingness within social media advertising scenarios.

### 5.7 Summary of effect sizes

Although previous analyses reveal that hypothesized direct effects, indirect effects, moderating effects and conditional indirect effects reach statistical significance, statistical significance alone cannot reflect the substantive strength of these relationships. Particularly given the relatively large sample size of this study, minor effects may achieve statistical significance. Therefore, this study further summarizes standardized path coefficients, proportions of mediating effects, incremental explained variance, and moderated mediation indices to evaluate the practical magnitude of each effect, as shown in Table 14.

**Table 14:** Summary of observed effect sizes.

Effect category	Path	Effect indicator	Observed value	Interpretation
Direct association	X1 → M1	Standardized β	0.312	Moderate
	X2 → M2	Standardized β	0.264	Small-to-moderate
	X3 → M3	Standardized *β*	0.345	Moderate
	M1 → Y1	Standardized *β*	0.276	Small-to-moderate
	M2 → Y2	Standardized β	0.290	Small-to-moderate
	M3 → Y3	Standardized β	0.325	Moderate
Mediation	X1 → M1 → Y1	Proportion mediated	28.7%	Partial mediation
	X2 → M2 → Y2	Proportion mediated	27.5%	Partial mediation
	X3 → M3 → Y3	Proportion mediated	31.3%	Partial mediation
Moderation	X1 × W1 → M1	ΔR^2^	0.018	Small incremental effect
	X2 × W1 → M2	ΔR^2^	0.014	Small incremental effect
	X3 × W1 → M3	ΔR^2^	0.022	Small incremental effect
	X1 × W2 → M1	ΔR^2^	0.016	Small incremental effect
	X2 × W2 → M2	ΔR^2^	0.012	Small incremental effect
	X3 × W2 → M3	ΔR^2^	0.019	Small incremental effect
Moderated mediation	X1 → M1 → Y1, moderated by W1	Index	0.033	Statistically detectable
	X2 → M2 → Y2, moderated by W1	Index	0.027	Statistically detectable
	X3 → M3 → Y3, moderated by W1	Index	0.047	Largest W1-related index
	X1 → M1 → Y1, moderated by W2	Index	0.028	Statistically detectable
	X2 → M2 → Y2, moderated by W2	Index	0.025	Smallest moderated mediation index
	X3 → M3 → Y3, moderated by W2	Index	0.043	Largest W2-related index

Standardized coefficients of 6 main relationships range from 0.264 to 0.345. These values indicate weak-to-moderate associations rather than strong effects. The three indirect effects account for 27.5 to 31.3% of the corresponding total effects, representing partial mediation. By contrast, incremental explained variance brought by interaction terms only ranges from 0.012 to 0.022. Thus, despite statistical significance, moderating effects have limited additional explanatory power. Moderated mediation indices fall between 0.025 and 0.047. Among them, perceived platform interactivity exerts the most prominent conditional effect on the path of emotional resonance—relatedness need fulfillment—advertising sharing intention. On the whole, the proposed model obtains statistical support in the current sample. Nevertheless, practical magnitudes of all effects, especially moderating effects, require cautious interpretation.

### 5.8 Robustness test and sensitivity analysis

To further test the stability of the research results, this study conducts robustness tests and sensitivity analyses, as follows:

- Based on the main model, age, education level, monthly income, city tier, social media usage frequency and primary advertising browsing platforms are added as control variables to re-estimate the three core mediating paths.
- After deleting the boundary samples with the lowest 5% of the answering time, the model is re-estimated to eliminate the potential impact of low-quality answers on the results.
- Group tests are conducted according to respondents’ primary advertising browsing platforms. The tests examine whether the directions of three core indirect paths remain consistent across subsamples of WeChat, Weibo, TikTok, and Xiaohongshu.
- The number of Bootstrap resamples is increased from 5,000 to 10,000 to test whether the indirect effect estimation results are stable
- The post-stratified weighting method is adopted to adjust the sample structure, thereby testing the sensitivity of the results to the sample structure.

The results are listed in Table 15.

**Table 15:** Robustness test and sensitivity analysis results.

**Indirect path**	**Moderator**	**Low level**	**Indirect effect at low level**	**Mean level**	**Indirect effect at mean level**
Advertising authenticity → autonomy need fulfillment → brand loyalty	Perceived platform interactivity (W1)	2.986	0.061	3.724	0.086
Advertising interactivity → competence need fulfillment → purchase intention	Perceived platform interactivity (W1)	2.986	0.052	3.724	0.077
Emotional resonance → relatedness need fulfillment → advertising sharing intention	Perceived platform interactivity (W1)	2.986	0.078	3.724	0.112
Advertising authenticity → autonomy need fulfillment → brand loyalty	Community belongingness (W2)	3.054	0.064	3.806	0.086
Advertising interactivity → competence need fulfillment → purchase intention	Community belongingness (W2)	3.054	0.055	3.806	0.077
Emotional resonance → relatedness need fulfillment → advertising sharing intention	Community belongingness (W2)	3.054	0.082	3.806	0.112

From Table 15, after adding control variables, deleting low-quality boundary samples, increasing the number of Bootstrap samples, and conducting post-stratified weighting, the directions and significance of the three core indirect paths remain stable. The results of the platform grouping test also show that the core path directions in different major advertising browsing platform samples are all positive and consistent. The above results indicate that the main conclusion of this study has good robustness.

### 5.9 Summary of hypothesis testing results

Based on the test results of the direct path, mediating effect, moderating effect, and moderated mediation effect mentioned above, this study summarizes the research hypotheses proposed in Section 3. The results are given in Table 16.

**Table 16:** Summary of hypothesis testing results.

Hypothesis	Tested relationship	Observed result	Effect-size information	Statistical judgment
H1a	X1 → M1	β = 0.312, p < 0.001	Moderate standardized association	Supported in the current sample
H1b	X2 → M2	β = 0.264, *p* < 0.01	Small-to-moderate standardized association	Supported in the current sample
H1c	X3 → M3	*β* = 0.345, *p* < 0.001	Moderate standardized association	Supported in the current sample
H2a	M1 → Y1	β = 0.276, p < 0.01	Small-to-moderate standardized association	Supported in the current sample
H2b	M2 → Y2	β = 0.290, p < 0.001	Small-to-moderate standardized association	Supported in the current sample
H2c	M3 → Y3	β = 0.325, *p* < 0.001	Moderate standardized association	Supported in the current sample
H3a	X1 → M1 → Y1	Indirect effect = 0.086, 95% CI [0.038, 0.141]	28.7% of total effect	Supported in the current sample
H3b	X2 → M2 → Y2	Indirect effect = 0.077, 95% CI [0.031, 0.128]	27.5% of total effect	Supported in the current sample
H3c	X3 → M3 → Y3	Indirect effect = 0.112, 95% CI [0.056, 0.174]	31.3% of total effect	Supported in the current sample
H4a	X1 × W1 → M1	β = 0.118, ΔR^2^ = 0.018	1.8% incremental variance	Supported; small incremental effect
H4b	X2 × W1 → M2	β = 0.092, ΔR^2^ = 0.014	1.4% incremental variance	Supported; small incremental effect
H4c	X3 × W1 → M3	β = 0.145, ΔR^2^ = 0.022	2.2% incremental variance	Supported; small incremental effect
H5a	X1 × W2 → M1	β = 0.102, ΔR^2^ = 0.016	1.6% incremental variance	Supported; small incremental effect
H5b	X2 × W2 → M2	β = 0.087, ΔR^2^ = 0.012	1.2% incremental variance	Supported; small incremental effect
H5c	X3 × W2 → M3	β = 0.131, ΔR^2^ = 0.019	1.9% incremental variance	Supported; small incremental effect
H6a	W1 moderates X1 → M1 → Y1	Index = 0.033, 95% CI [0.012, 0.061]	Low-to-high increase = 0.053	Supported in the current sample
H6b	W1 moderates X2 → M2 → Y2	Index = 0.027, 95% CI [0.009, 0.052]	Low-to-high increase = 0.049	Supported in the current sample
H6c	W1 moderates X3 → M3 → Y3	Index = 0.047, 95% CI [0.019, 0.083]	Low-to-high increase = 0.073	Supported in the current sample
H7a	W2 moderates X1 → M1 → Y1	Index = 0.028, 95% CI [0.010, 0.055]	Low-to-high increase = 0.045	Supported in the current sample
H7b	W2 moderates X2 → M2 → Y2	Index = 0.025, 95% CI [0.007, 0.049]	Low-to-high increase = 0.042	Supported in the current sample
H7c	W2 moderates X3 → M3 → Y3	Index = 0.043, 95% CI [0.016, 0.078]	Low-to-high increase = 0.064	Supported in the current sample

Table 16 indicates that all direct path hypotheses, mediation hypotheses, moderation hypotheses, and moderated mediation hypotheses proposed in this study receive full empirical support. Specifically, advertising authenticity, interactivity, and emotional resonance each have significant positive correlations with autonomy, competence, and belonging, respectively. Autonomy, competence, and belonging further form significant positive associations with brand loyalty, purchase intention, and information dissemination willingness. Mediation tests confirm that basic psychological need fulfillment plays a significant mediating role between advertising content characteristics and advertising effectiveness. Results from moderation and moderated mediation effect tests further illustrate that platform interactivity and community belongingness strengthen the correlations between advertising content characteristics and psychological need fulfillment, further amplifying the corresponding indirect relationships.

## 6 Conclusion

Based on the revised individual-level moderated mediation model, this study examines the relationship among social media advertising content characteristics, basic psychological need fulfillment, perceived platform environment, and advertising effectiveness. The results show that advertising authenticity, interactivity, and emotional resonance are significantly positively associated with autonomy, competence, and belonging, respectively. Meanwhile, it further forms indirect associations with brand loyalty, purchase intention, and information dissemination willingness through different psychological need paths. Specifically, authenticity is indirectly associated with brand loyalty through autonomy fulfillment; interactivity forms an indirect association with purchase intention via competence fulfillment; emotional resonance is indirectly related to information dissemination willingness through belonging fulfillment. Among them, the indirect effect of the emotional resonance—belonging—information dissemination willingness path is relatively stronger. This indicates that emotional connection, value recognition, and group belonging experience are important psychological conditions for promoting users to actively spread advertising content.

The main value of this study lies in verifying the correlation between advertising content characteristics and advertising effectiveness; it also further reveals the psychological mechanism therein. Studies show that advertising content is not directly and solely associated with advertising effectiveness. Instead, indirect paths are formed by satisfying users’ basic psychological needs such as autonomy, competence, and belonging. Therefore, the formation of social media advertising effectiveness is the result of the combined effect of advertising content characteristics, user psychological need fulfillment, and perceived platform environments.

Meanwhile, this study finds that platform interactivity and community belongingness can positively moderate the relationship between advertising content characteristics and basic psychological need fulfillment. When users perceive strong platform interactivity and high community belongingness, advertising authenticity, interactivity, and emotional resonance are more likely to form a positive association with the corresponding psychological need fulfillment. At the same time, it further enhances the indirect relationship between it and advertising effectiveness. This indicates that the platform environment is not merely a simple background for dissemination. Rather, it is an important situational condition that affects the transformation of advertising content characteristics into psychological need fulfillment and advertising effectiveness.

The theoretical contribution of this study mainly lies in constructing an individual-level analytical framework based on SDT and testing three specific association paths in a sample of female social media users. The results show that autonomy, competence, and relatedness need fulfillment play a statistical mediating role between specific advertising content characteristics and advertising effect variables. User-perceived platform interactivity and community belongingness are also related to the above-mentioned differences in relationship strength. However, this study does not prove that these mechanisms are unique to female users, nor can it confirm the effect or strict causal process at the objective platform level.

In terms of practical implications, the conclusions of this study are limited to the scope supported by empirical data. These results show that advertising content perceived as authentic, credible, and transparent by users more easily satisfies their autonomy needs and generates positive correlations with brand loyalty. Advertising content that provides opportunities for participation, feedback, commenting, sharing, and communication better fulfills users’ competence needs and forms positive links with purchase intention. When advertising content can resonate with users’ emotions, life experiences, value recognition, or self-perception, it is easier to meet their belonging needs; in addition, it creates positive associations with information dissemination willingness. Therefore, in the scope of the current sample and research design, the results suggest that advertising practitioners can focus on users’ perceptions of content authenticity, opportunities for meaningful interaction, and emotional relevance. This study carries out no experimental manipulation on advertising content and does not track users’ actual behavioral outcomes; therefore, the above conclusions can only be used as preliminary references for advertising content design and should not be interpreted as causally verified production rules.

This study still has certain limitations. First, this study uses cross-sectional self-reported questionnaire data and cannot conduct strict causal inference. Second, the sample is derived from a limited number of online voluntary responses, and responses are restricted to female social media users aged 18 to 35. Therefore, caution should be exercised in promotion. Third, this study does not directly measure variables (such as advertising fatigue, perceived manipulation, push frequency, algorithm transparency, and privacy worries). Thus, no empirical conclusions are drawn on these factors. Future research can combine longitudinal tracking data, experimental design, and platform behavior data to further examine the dynamic relationship among advertising content, psychological needs, platform environments, and algorithmic recommendation mechanisms.

## Data Availability

The raw data supporting the conclusions of this article will be made available by the authors, without undue reservation.
